# Ten-Year Evaluation of Ventilator-Associated Pneumonia (VAP) According to Initial Empiric Treatment: A Retrospective Analysis Using Real-World Data

**DOI:** 10.3390/biomedicines13020360

**Published:** 2025-02-05

**Authors:** Alejandro Rodríguez, Julen Berrueta, Carolina Páez, Ronny Huertas, Marco Marotta, Laura Claverias, Josep Gómez, Sandra Trefler, Frederic F. Gómez Bertomeu, María Dolores Guerrero-Torres, Sergio Pardo-Granell, Ester Picó-Plana, Alicia Selles-Sánchez, Francisco Javier Candel, Ignacio Martín-Loeches, María Bodí

**Affiliations:** 1 Intensive Care Department, Hospital Universitari Joan XXIII, 43005 Tarragona, Spain; julen.berrueta@estudiants.urv.cat (J.B.); mmarottapais@gmail.com (M.M.); lauraclaverias@gmail.com (L.C.); sitrefler@yahoo.es (S.T.); mbodi.hj23.ics@gencat.cat (M.B.); 2 IISPV (Instituto de Investigación Sanitaria Pere Virgili), 43005 Tarragona, Spain; ffgomez.hj23.ics@gencat.cat; 3 Centre for Biomedical Research Network Respiratory Diseases (CIBERES), 43005 Tarragona, Spain; 4 Faculty of Medicine and Health Sciences, Department of Pharmacology, Rovira & Virgili University, 43201 Reus, Spain; 5 Tarragona Health Data Research Working Group (THeDaR), 43005 Tarragona, Spain; ronnyghg@gmail.com; 6 Postgrado Medicina Crítica y Cuidado Intensivo, Facultad de Medicina, Fundación Universitari Ciencias de la Salud, Distrito Especial, Cra. 54 No. 67A-80, Bogotá 111221, Colombia; ncpaez@fucsalud.edu.co; 7 Technical Secretary’s Department, Hospital Universitari Joan XXIII, 43005 Tarragona, Spain; josep.goal@gmail.com; 8 Microbiology/Clinical Analysis Laboratory, Hospital Universitari de Tarragona Joan XXIII, 43005 Tarragon, Spain; maguerrero.hj23.ics@gencat.cat (M.D.G.-T.); separdo.hj23.ics@gencat.cat (S.P.-G.); eplana.hj23.ics@gencat.cat (E.P.-P.); aselles.hj23.ics@gencat.cat (A.S.-S.); 9 Faculty of Medicine and Health Sciences, Department of Medicine and Surgery, Rovira & Virgili University, 43005 Tarragona, Spain; 10 Centre for Biomedical Research in Infectious Diseases Network (CIBERINFEC), 28220 Madrid, Spain; 11 Clinical Microbiology & Infectious Diseases Department, Hospital Clínico Universitario San Carlos, 28040 Madrid, Spain; fj.candel@gmail.com; 12 San Carlos Hospital Health Research Institutes (IdISSC & IML), 28040 Madrid, Spain; 13 Department of Intensive Care Medicine, Multidisciplinary Intensive Care Research Organization (MICRO), St James’ Hospital, D08 NHY1 Dublin, Ireland; drmartinloeches@gmail.com

**Keywords:** ventilator-associated pneumonia, empiric antibiotic treatment, outcome, epidemiology, syndromic respiratory panel

## Abstract

**Background/Objectives**: Ventilator-associated pneumonia (VAP) is the main nosocomial infection in intensive care units (ICUs) that causes the highest morbidity and mortality. The aim of our study is to investigate variations in crude ICU mortality among patients with VAP over the past decade. We also wish to identify associated risk factors, evaluate changes in the etiology, and assess the incidence and impact of inappropriate empirical antibiotic treatment (IEAT). **Methods**: We conducted a retrospective, observational, single-center study over a 10-year period (2014–2024), including critically ill patients who developed VAP. The population was divided into three periods: (P1) from 2014 to 2018 (pre-COVID-19); (P2) from 2019 to 2021 (COVID-19); and (P3) from 2022 to 2024 (post-COVID-19). Binary logistic regression was used to identify which variables were independently associated with ICU mortality. **Results**: A total of 220 patients were included in the study (P1 = 47, P2 = 96, and P3 = 77 patients). The most prevalent microorganisms identified were *P. aeruginosa*, *Klebsiella* spp., and *S. aureus*. Significant variations in etiology were not observed over the years. The incidence of IEAT was 4.5%, with no observed differences between the study periods. Crude ICU mortality was 33.6%, with higher rates observed in IEAT (40% vs. 33.3%, *p* = 0.73). In patients with appropriate empiric antibiotic treatment (AEAT), there was a significant decrease in crude mortality over the years from 42.2% in P1 to 22.2% in P3 (*p* < 0.001). Age (OR = 1.04; 95% CI = 1.01–1.08) and P2 (OR = 2.8; 95% CI = 1.1–7.4) were found to be independently associated with an increased risk of mortality. Conversely, a lower risk of death was associated with mean arterial pressure (OR = 0.94; 95% CI = 0.69–0.99) and the use of syndromic respiratory panel (OR = 0.23; 95% CI = 0.07–0.68). **Conclusions**: A reduction in crude VAP mortality over the years was observed, with no change in the etiology or rate of IEAT. The implementation of protocols using respiratory syndromic panels could be a measure to implement to reduce VAP mortality.

## 1. Introduction

Ventilator-associated pneumonia (VAP) is the leading nosocomial infection in intensive care units (ICUs) [1]. VAP is associated with more days of invasive mechanical ventilation, increased antibiotic consumption, and higher hospital costs [2]. However, the association of VAP with increased mortality remains controversial [3]. This complication affects 8–28% of patients undergoing mechanical ventilation [1], with large variations depending on the country, type of ICU, and criteria used to identify VAP [4,5].

In older series, the most common pathogens recovered from bronchoscopy specimens were *Pseudomonas aeruginosa* (24.4%), *Staphylococcus aureus* (20.4%), and *Enterobacterales* (14.1%) [1]. Several studies have shown that the initial treatment of VAP with inadequate antimicrobial agents is associated with a worse prognosis [6,7]. It is therefore particularly important to understand the epidemiology and resistance trends of the bacteria involved.

The last decade has seen significant changes both in the respiratory management of mechanically ventilated patients, especially those affected by VAP [8,9], and in the epidemiology of multidrug-resistant (MDR) bacteria in Europe [10,11], especially after the SARS-CoV-2 pandemic, where a significant increase in resistance among Gram-negative bacilli was observed [12,13]. Both conditions may affect the outcome of critically ill patients on mechanical ventilation.

The National Healthcare Safety Network of the Centers for Disease Control and Prevention (CDC) has reported large declines in the incidence of VAP in medical and surgical ICUs over the past 15 years [14]. However, these results were not confirmed by an analysis using a stable definition of VAP conducted by the Medicare Patient Safety Monitoring System between 2005 and 2013 [15]. However, other authors [16] in countries where there is no financial penalty associated with infectious complications have shown a reduction in the incidence of VAP over the years.

We hypothesize that changes in the management of ventilated patients and the evolving epidemiology of multidrug-resistant bacteria, particularly in the post-SARS-CoV-2 era, have influenced ICU mortality in patients with VAP. To address this, our study aims to investigate variations in crude ICU mortality among patients with VAP over the past decade. Furthermore, we seek to identify associated risk factors for mortality, evaluate changes in the microbiological etiology of VAP, and assess the incidence and impact of inappropriate empirical antibiotic treatment (IEAT). These findings will provide critical insights to inform future strategies for improving patient outcomes in the ICU.

## 2. Materials and Methods

### 2.1. Study Design and Participants

A retrospective, observational, single-center study was conducted over a 10-year period (2014–2024), including critically ill patients (≥18 years of age) consecutively admitted to a 30-bed ICU. The analysis included all critically ill patients who developed VAP with confirmed microbiology. Only bacterial VAP was considered; patients with fungal etiologies were excluded. Furthermore, only the first episode of microbiologically confirmed VAP per patient was included in this analysis. During the 10-year study period, the population was divided into three periods. The first period was from 2014 to 2018 (pre-COVID-19 period). The second period, from 2019 to 2021, coincided with the SARS-CoV-2 pandemic (COVID-19 period), and the third period ranged from 2022 to 2024 (post-COVID-19 period). (Supplementary Figure S1).

### 2.2. Variables

The variables studied are shown in Table 1. These variables were obtained automatically from the clinical information system (CIS, Centricity Critical Care^®^, GE HealthCare, Germany) through the development of ETL (extract, transform, and load) specifically created using SQL and Python. The CIS automatically records all data from devices connected to the patient every two minutes, including hemodynamic variables, hourly diuresis, clinical parameters, and laboratory values, as well as information on medication administered. In addition, healthcare professionals register all patient-related information throughout the ICU stay (VAP). Demographic, comorbidity, clinical, and laboratory variables on admission to the ICU were obtained. We also calculated the number of days of mechanical ventilation and evolution in the ICU.

### 2.3. Definitions

VAP was defined as a respiratory infection occurring in mechanically ventilated patients according to the guidelines of the European Respiratory Society (ERS), the European Society of Intensive Care Medicine (ESICM), the European Society of Clinical Microbiology and Infectious Diseases (ESCMID), and the Asociación Latinoamericana del Tórax (ALAT) [17]. VAP was defined as pneumonia occurring more than 48 h after endotracheal intubation with fever, without other apparent causes, with new or increased sputum production, positive endotracheal aspirate (ETA) culture (>10^6^ CFU/mL), or bronchoalveolar lavage (BAL) culture (>10^4^ CFU/mL), with at least one respiratory pathogen known to cause pneumonia, and with radiographic evidence of nosocomial pneumonia.

Empirical antimicrobials were selected based on specialist clinical judgment and internal ICU protocols, which could subsequently be modified by the ASP (antimicrobial stewardship program) team, based on the clinical response in the days following VAP diagnosis or final microbiology results.

During the entire study period, the same bundles of VAP prevention measures recommended by the Spanish Society of Intensive Care Medicine and Coronary Units (SEMICYUC) within the Pneumonia zero program were applied. Our ICU does not use digestive decontamination in ventilated patients. From 2019, a rapid microbiological diagnostic protocol based on the Biofire© Filmarray Pneumonia Panel Plus© (bioMérieux, Marcy-l’Étoile, France) was implemented in all patients with suspected multi-drug resistant (MDR) microorganisms, as previously published [18]. From 2014 to 2019, the treatment protocol in our ICU included the administration of an antipseudomonal agent: carbapenem (meropenem) or piperacillin/tazobactam, with or without another agent with activity against resistant cocci (linezolid or tigecycline). In patients with no apparent risk for *Pseudomonas aeruginosa*, ertapenem was the empirical treatment option accepted by our ICU protocol.

As of 2019, due to the observed high resistance of carbapenem and piperacillin/tazobactam (>40%) to *Pseudomonas aeruginosa*, empirical treatment was changed to aztreonam or ceftolozane/tazobactam or ceftazidime/avibactam according to the sensitivity reported by the microbiology service.

Empirical antibiotic therapy was considered appropriate (AEAT) if the bacteria detected in vitro were susceptible to at least one antibiotic administered. Empirical antibiotic therapy was considered inappropriate (IEAT) if the isolate did not show sensitivity. Multidrug resistance (MDR) was defined as acquired non-susceptibility to at least one agent in three or more antimicrobial categories, and extensively drug resistant (XDR) was defined as non-susceptibility to at least one agent in all but two or fewer antimicrobial categories (i.e., bacterial isolates remain susceptible to only one or two categories) [19].

### 2.4. Objectives and Follow-Up

#### 2.4.1. Primary Objective

The primary objective was to assess variations in crude ICU mortality among patients with VAP over a 10-year observation period and to identify associated risk factors.

#### 2.4.2. Secondary Objectives

To evaluate potential variations in the etiology of VAP.

To assess the incidence of IEAT, the associated microorganisms, and their impact on mortality.

#### 2.4.3. Primary Outcome

The primary outcome was all-cause mortality in the ICU. Patients discharged alive from the ICU were considered survivors, while mortality was defined as any death occurring during the ICU admission.

#### 2.4.4. Follow-Up

All complications and outcomes were monitored throughout the ICU admission to provide a comprehensive understanding of patient trajectories and outcomes

### 2.5. Statistical Analysis

Prior to the study, we did not perform any statistical calculation of sample size. Instead, we selected the sample size to be equal to the number of patients admitted to the ICUs of the participants with microbiologically confirmed VAP during the study period. To provide an overview of the baseline characteristics, the continuous variables were expressed as a median (Q1–Q3 = IQR), while the categorical variables were expressed as the number of cases (percentage). For demographic and clinical characteristics, differences between groups were assessed using the following tests: chi-squared test and Fisher’s exact test or “*t*” test for categorical variables and Mann–Whitney U test for continuous variables.

Binary logistic regression was used to identify which variables were independently associated with ICU mortality. All variables with a statistical significance (*p* < 0.05) in the bivariate comparison between groups were included in the GLM (generalized linear model). In addition, period 2 coinciding with the COVID-19 pandemic and the implementation of a protocol for rapid microbiological diagnosis using molecular techniques were incorporated into the final model, as they are the factors of clinical interest that may be related to the evolution of patients despite not reaching statistical significance in the comparative table.

To validate the regression model, the population was randomly divided into a training group (70% of the population) and a validation group (30% of the population) through stratifying by ICU mortality. The results are presented as odds ratios (OR) with 95% confidence intervals (95% CI). Values below 0.05 were considered significant.

The model’s performance was evaluated based on its accuracy, precision, sensitivity, specificity, and area under (AUC) the receiver operating characteristic (ROC) curve. Furthermore, the Hosmer–Lemeshow goodness of fit was determined, and the presence of collinearity between the explanatory variables was examined using variance inflation factors (VIFs) [20]. To check whether the linear functional form of the regression model is correct, the Ramsey regression equation specification error test (RESET) was applied. It checks for overlooked variables, incorrect functional forms, or omitted non-linearities by adding terms constructed as powers or products of the fitted values and assessing their significance [21]. Furthermore, we conducted k-fold cross-validation (K = 10), which entailed partitioning the original data into two distinct sets: a primary development set (Train) and a secondary validation set (Test). The training set is then divided into k subsets. During the training process, each k subset is used as the model test set, while the remaining data are used as the training set. Once the required number of iterations has been completed, the accuracy and error rates are calculated for each of the models produced. The final accuracy and error rates are then obtained by averaging the k-trained models.

The statistical analyses were performed using R software (version 4.4.2 for Windows).

## 3. Results

### 3.1. General

A total of 227 patients met the eligibility criteria. The initial empiric treatment was administered to 90 patients (39.6%) who received meropenem (MRP), 79 (34.8%) received piperacillin/tazobactam (PTZ), 51 (22.5%) received ertapenem (ETP), and only 7 patients received alternative treatments (aztreonam = 2; cloxacillin = 2; ceftriaxone = 1; fluoroquinolone = 2). For the purposes of this analysis, only the three majority groups were considered, resulting in a final sample size of 220 patients with VAP (Supplementary Figure S1). Only 2 patients had a hospital admission (non-ICU) within the 3 months prior to this admission.

Over the course of the 10-year study, the population was divided into three periods. The first period included 47 patients, with an incidence density (ID) of 2.8 episodes of VAP per 1000 ventilator days (MV). The second period coincided with the SARS-CoV-2 pandemic and included 96 patients with an incidence density of 4.9 episodes per 1000 ventilator days. The third period included 77 patients with an incidence density of 3.4 episodes per 1000 days of mechanical ventilation. Please refer to Supplementary Figure S2 for a visual representation of the annual incidence in each period.

The first period cohort exhibited elevated levels of organ dysfunction (SOFA) and inflammation (CRP), while displaying a reduced incidence of medical admissions. In contrast, there was a higher prevalence of hypertension and immunosuppression in the second and third periods. Please refer to Table 1 for a comprehensive overview of the patients’ characteristics across all three periods.

### 3.2. Etiology of VAP over the Years

The most frequently isolated microorganisms were Gram-negative bacilli (GNB). *Pseudomonas aeruginosa* and *Klebsiella* spp. were the most frequently isolated GNB across all periods (see Table 2). There was an upward trend in the isolation of these microorganisms in periods 2 and 3, although this was not statistically significant. Similarly, there was no significant change observed in the isolation of Gram-positive cocci. Methicillin-resistant *Staphylococcus aureus* and *Acinetobacter* spp. isolates were anecdotal in all three periods. *Stenotrophomonas maltophilia* was more frequent in period 1 than in the other periods, but without significant differences.

A total of 69 microorganisms were isolated from the 47 patients in period 1. Of these, 22 patients (47%) had two microorganisms isolated and 6 patients (12.7%) had three microorganisms isolated. In period 2, a total of 166 microorganisms were isolated in 96 patients. Among them, 39 patients (56.5%) had two microorganisms isolated, while 16 patients (16.6%) had three microorganisms isolated. Finally, in period 3, a total of 130 microorganisms were isolated in 77 patients. Among them, 28 (36.3%) had two microorganisms isolated, while 13 (16.8%) has three microorganisms isolated.

### 3.3. Empiric Antibiotic Treatment

Of the 220 patients, 75% (*n* = 165) received empiric antibiotics as monotherapy. In the remaining 25% (*n* = 55), the second antimicrobial was linezolid (*n* = 50, 91%) or tigecycline (*n* = 5, 9%).

The overall incidence of inappropriate empirical antibiotic treatment (IEAT) was 4.5% (10/220), with no differences between the study periods: 4.3% (*n* = 2), 3.1% (*n* = 3), and 6.5% (*n* = 5) for periods 1, 2, and 3, respectively. Furthermore, there was no discernible difference in the incidence of IEAT according to the antimicrobial agent used, whether ertapenem (*n* = 2; 3.9%), meropenem (*n* = 3; 3.3%), or piperacillin/tazobactam (*n* = 5; 6.3%). *Stenotrophomonas maltophilia* and *Pseudomonas aeruginosa* MDR were the microorganisms most commonly associated with IEAT (Supplementary Table S1).

Of the 210 patients with appropriate empirical antibiotic treatment, 150 (71.4%) had their treatment adjusted. In 138 (92%) de-escalation was realized and in 12 (8%) the number of antibiotics was reduced. This adjustment did not differ between periods 1 (77.8%) and 2 (66.7%, *p* = 0.18), but was significantly lower in period 3 (56.9%, *p* < 0.05).

### 3.4. ICU Mortality Rate

Crude ICU mortality in the whole population was 33.6% (*n* = 74). While patients with inadequate empirical antimicrobial treatment (IEAT) demonstrated a higher mortality rate of 40% (*n* = 4) compared to those who received adequate treatment (33.3%, *n* = 70), but this difference was not statistically significant (*p* = 0.73). In patients with appropriate empirical antibiotics, crude ICU mortality decreased significantly over the years from 42.2% (19/95) in period 1 and 37.6% (35/93) in period 2 to 22.2% (16/72) in period 3 (*p* < 0.001). In particular, a significant decrease was observed in period 3 compared to period 1 (*p* = 0.02) and period 2 (*p* = 0.03).

Although patients receiving meropenem as adequate empirical treatment had lower mortality (29.9%, 26/87) than those receiving piperacillin/tazobactam (37.8%, 28/74, *p* = 0.28) or ertapenem (32.7%, 16/49, *p* = 0.30), these differences did not reach significance.

Patients who did not survive were older, had a higher level of organ dysfunction as measured by the SOFA score, a higher frequency of chronic heart disease, a lower SO_2_/FiO_2_ ratio, a higher frequency of hypotension, and a reduced diuresis volume (Table 3).

#### Lineal Regression Analysis (GLM)

To study the impact of variables on mortality, a multivariate regression model (GLM) was developed. The population was randomly divided into a training subset, with 70% of the population (*n* = 154), and a test subset, with the remaining 30% (*n* = 66).

The dependent variable was ICU mortality, while the covariates included were age, sex, chronic heart disease, bacteremia, inappropriate antibiotic treatment, period 2, PCT, SOFA score, mean arterial pressure, SO_2_/FiO_2_, serum creatinine, COVID-19 period, and filmarrays protocol. All covariates were considered on the day of diagnosis of VAP.

**Figure 1:**
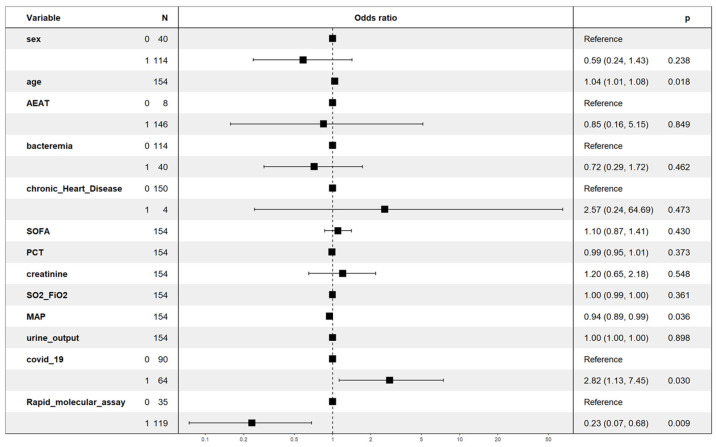
Variables included in the multivariate regression model (GLM) and corresponding odds ratios (OR) in train subset. (AEAT: appropriate empiric antibiotic treatment; SOFA: sequential organ failure assessment; PCT: procalcitonin; SO_2_: oxygen saturation; FiO_2_: fraction of inspired oxygen; MAP: mean arterial pressure.)

Only age with an OR = 1.04 (95% CI = 1.01–1.08) for each year and COVID-19 period (P2) with an OR = 2.8 (95% CI = 1.1–7.4) were independently associated with an increased risk of mortality. In contrast, mean arterial pressure with OR = 0.94 (95% CI = 0.69–0.99) and the use of rapid microbiological diagnosis with Biofire^®^ Filmarray Pneumonia with OR = 0.23 (95% CI = 0.07–0.68) were associated with a lower risk of death. Period 3 had high collinearity with a VIF greater than 15 and had to be removed from the model (Figure 1).

The internal validation of the model showed an accuracy of 74% with a sensitivity of 63% and a high specificity of 80% with a positive predictive value of 55% and a negative predictive value of 84%. The Hosmer–Lemeshow test was *p* = 0.37, showing no difference between the predicted and observed values, and the RESET linearity test was *p* = 0.12, showing linearity in the model. Finally, the AUC was 0.76 (0.64–0.88) (Supplementary Figure S3). Cross-validation (K = 10) did not improve model performance, but did maintain an accuracy of 74% (Supplementary Table S2).

## 4. Discussion

Our main finding was that the crude ICU mortality of VAP decreased significantly over the years, with a significant reduction in period 3 (2022–2024). This reduction was observed despite no significant change in etiology and no difference in empirical antibiotic treatment between the controlled periods. Conditions associated with crude ICU mortality were patient age and period 2, which coincided with the COVID-19 pandemic. On the other hand, mean arterial pressure and, in particular, the implementation of a rapid diagnostic protocol based on molecular techniques were associated with a lower mortality risk.

Although crude ICU mortality associated with VAP has been reported to be as high as 50% [22], there remains considerable controversy about the extent to which VAP contributes to death in ICU patients. In contrast, VAP has been consistently associated with prolonged mechanical ventilation and ICU stay. In our study, crude mortality decreased significantly over the years from 44.7% to 22.1%. Similarly, de Miguel-Díez et al. [16] demonstrated, in an elegant study of a large administrative database including more than 9000 patients, that crude mortality decreased significantly over the study period, from 35.74% in 2010 to 32.81% in 2014. Although mortality decreased by only 2.9%, this difference is significant due to the large number of patients included, so the clinical impact of this decrease appears to be very small. On the other hand, this mortality behavior was not observed by Fihmana et al. [23], who did not find a decrease in mortality when studying patients with VAP during the period 2007–2011, with incidences of 37%, 37%, 27%, 31%, and 36% for the 5 years studied.

The strong association of P2 with mortality in the multivariate model may be related to the COVID-19 pandemic during almost all of P2. A recent European study [24] found that the incidence of VAP was higher in patients with SARS-CoV-2 pneumonia (36.1%) than in those with influenza pneumonia (22.2%) or no viral infection at the time of ICU admission (16.5%). These data are consistent with our findings, as an increased incidence density was observed in P2. Furthermore, the authors reported that VAP was associated with a significantly increased risk of 28-day mortality in the ARDS-CoV-2 group (adjusted HR: 1.65 [95% CI: 1.11–2.46]), but not in the influenza (1.74 [0.99–3.06]) or non-viral infection (1.13 [0.68–1.86]) groups. As suggested by Rouzé et al. [25], possible explanations for the high incidence of VAP in COVID-19 patients include the long duration of invasive mechanical ventilation, the high incidence of acute respiratory distress syndrome (ARDS), and immunosuppressive treatment. In our study, the number of days on mechanical ventilation did not differ between P2 and P3. In addition, we did not determine how many patients had ARDS before the development of VAP (see limitations). Finally, we did not observe significant differences in immunocompromised patients between P2 and P3. Therefore, we can only hypothesize that this association may be related to COVID-19 and other confounders not assessed in our model.

Our study was not designed to assess the attributable mortality of VAP, so several factors that may influence the outcome should be discussed. The first of these is empirical antimicrobial treatment. Our results show that there is a low percentage of IEAT in all periods, which may be due to empirical treatment with broad-spectrum agents, possibly more than indicated. This may explain the high rate of de-escalation observed in all periods. Although early reports observed a strong association between IEAT and mortality [6,26,27,28], more recent studies have questioned this association [29]. In our study, IEAT was not found to be a factor associated with mortality in multivariate analysis, in contrast to other reports [6,28]. The low incidence of IEAT observed may be related to the lack of significance of the 6% increase in mortality observed in patients with IEAT.

In addition, the type of empirical treatment should be considered, with meropenem, piperacillin/tazobactam, and ertapenem being the most commonly used drugs. Although unusual, our protocol includes the option of ertapenem if *Pseudomonas aeruginosa* is not suspected or proven. Ertapenem is a carbapenem with antimicrobial activity against most Gram-positive and Gram-negative aerobic and anaerobic bacterial infections, including ESBL-producing pathogens. It is a valid option when it is not desired to put pressure on the development of *Pseudomonas* spp. resistance. In addition, ertapenem has demonstrated efficacy in the clinical and microbiological resolution of VAP [30]. However, alterations in the maximum (or peak) serum concentration (C_max_) and AUC_0–last_ of ertapenem have been reported in critically ill patients related to critical pathology and hypo-albuminemia [31]. Bosselli et al. [32] observed satisfactory results, with the unbound ertapenem concentrations in both serum and pulmonary epithelial lining fluid (approximately 40% of the serum value) exceeding the MIC_90_ values for most pathogens found in early-onset VAP for 50–100% of the time.

In our study, we did not observe a higher incidence of IEAT in ertapenem patients, nor differences in crude mortality in this subgroup of patients compared to the meropenem or piperacillin/tazobactam. Since pharmacokinetic/pharmacodynamic (PK/PD) disturbances related to the volume of distribution and hypo-albuminemia can be partly addressed with increased dosing frequency, our protocol envisages the administration of 1 g every 12 h in patients with VAP.

Another point of interest is the variation in etiology. The microorganisms associated with VAP vary according to many factors, such as the duration of mechanical ventilation, the length of hospital and intensive care unit stays prior to VAP, the timing and cumulative antimicrobial exposure, the local ecology, the occurrence of potential epidemic phenomena, and, most importantly, the local antimicrobial policy that exerts pressure on the development of resistance.

Gram-negative organisms commonly implicated in VAP are *Pseudomonas aeruginosa*, *Escherichia coli*, *Klebsiella pneumoniae,* and *Acinetobacter* spp.; *Staphylococcus aureus* is the primary Gram-positive organism [33,34]. However, there is large variability related to local conditions. Our ICU is free of *Acinetobacter baumannii* and methicillin-resistant *S. aureus*, and the antimicrobial stewardship program (ASP) team applies a strict antimicrobial policy. Various authors [35,36] have reported considerable variation in the etiology of VAP, with an increasing number of MDR microorganisms being isolated over the years. However, this epidemiological behavior was not observed in our study, as also reported by de Miguel-Díez [16] et al.

It is important to note that, as suggested by Barbier et al. [37], the 63% of complications related to mechanical ventilation-associated infections were neither VAP nor due to a documented infection in the ICU, indicating that efforts should focus on the diagnostic strategy, using carbapenems only in patients with true infection and withholding carbapenems when the likelihood of infection is low. In this context, it is important to highlight the impact on mortality seen with the implementation of a rapid microbiological diagnostic protocol. Although our study cannot establish a causal relationship between mortality and the use of the rapid diagnostic protocol, multivariate analysis showed a strong and independent association between the two conditions. Our group [18] already reported the impact that the implementation of a rapid microbiological diagnostic protocol had on the reduction in meropenem and piperacillin/tazobactam resistance, as well as on the decrease in antimicrobial consumption measured in defined daily doses (DDD) in the whole ICU. The present study’s findings confirm the favorable impact of implementing protocols that include molecular microbiological diagnosis for the optimization of antimicrobial therapy.

Our study has the strength of being a 10-year analysis of the evolution of VAP using automatically generated real-world data. However, it has several limitations that must be taken into account.

First, it is a longitudinal but single-center study, so the results cannot be extrapolated to other centers.

Secondly, we did not design a study to assess attributable mortality with VAP, as our aim was to assess crude mortality over the 10 years. Different methods have been used to calculate attributable mortality in VAP, with estimates ranging from 0 to 60% [3]. Quantifying the impact of this condition on patient outcomes is also complicated by the time-dependent nature of the disease, which may include time-dependent bias, and by the fact that mortality and ICU discharge act as competing endpoints [3]. Given the difficulties and conflicting results of the studies on attributable mortality, our aim was to assess which factors affect crude mortality as a ‘hard’ outcome variable, rather than using complex statistical methods.

Thirdly, the development of acute respiratory distress syndrome may favor ventilator-associated pneumonia (VAP). This has been clearly demonstrated during the COVID-19 pandemic. Although the increased incidence density of VAP observed in P2 (which includes cases of SARS-CoV-2) could be justified by this cause, we can only hypothesize about this, since we have no data to determine or classify which patients have developed ARDS prior to VAP. However, the main objective of the study was to assess the mortality of patients who develop VAP.

Finally, we do not differentiate between early and late VAP. It is generally recognized that early-onset VAP (within the first 5–7 days of mechanical ventilation) in previously healthy patients not receiving antibiotics usually involves the isolation of microorganisms from the oropharynx without resistance mechanisms. In contrast, late-onset VAP (>5–7 days of ventilation) and VAP in patients with risk factors for multidrug-resistant (MDR) pathogens are more likely to be due to pathogens with different resistance mechanisms [22]. However, MDR pathogens can be isolated in early-onset VAP, mainly in the presence of certain risk factors, such as exposure to antimicrobials in the previous 90 days, and some authors have found comparable rates of MDR pathogens in patients with early- versus late-onset VAP [38,39,40].

## 5. Conclusions

Our study shows a reduction in crude VAP mortality over the years, with no change in VAP incidence, etiology, or inadequate empirical antibiotic treatment, and irrespective of the choice of initial antimicrobial agent. The favorable effect on mortality observed with the implementation of a rapid microbiological diagnostic protocol suggests that this measure should be carefully considered by ASP teams for implementation. However, our results should be interpreted with caution and confirmed by studies in other units.

## Figures and Tables

**Table 1:** Characteristics of 220 critically ill patients with ventilator-associated pneumonia according to study periods.

Variable	1st Period (n = 47)	2nd Period (96)	3rd Period (n = 77)	*p*-Value
General				
Age median (IQR) year	60 (50–68)	64(52–73)	60(49–69)	0.16
Male, *n* (%)	38 (80.9%)	69(71.9)	59(76.6)	0.48
SOFA score, median (IQR)	8 (6–10)	4(3–6)	4(2–7)	0.40
Medical disease, *n* (%)	27(57.4)	82(85.4)	54(70.1)	0.001
Comorbidities				
Diabetes mellitus, *n* (%)	5 (10.6)	24(25.0)	20(26.0)	0.09
Cirrhosis, *n* (%)	3(6.4)	4(4.2)	9(11.2)	0.16
Hypertension. *n* (%)	13(27.7)	56(58.3)	30(39.0)	0.001
Chronic heart disease, *n* (%)	3(6.4)	3(3.1)	0 (0.0)	0.08
Immunodepression, *n* (%)	0 (0.0)	8(8.3)	11(14.3)	0.01
Chronic obstructive pulmonary disease, *n* (%)	6(12.7)	11(11.4)	10(12.9)	0.80
Laboratory at ICU admission				
Hemoglobin, median (IQR) g/L	8.8(7.5–9.6)	9.3(7.9–10.5)	8.7(7.7–9.9)	0.14
White blood cells count, median (IQR) 10 × 9	17.0(10.9–22.6)	15.1(10.5–18.7)	14.4(9.4–21.7)	0.35
Lymphocytes, median (IQR) 10 × 9	0.9(0.6–1.4)	0.9(0.6–1.5)	0.9(0.6–1.26)	0.69
C-reactive protein, median (IQR) mg/dL	33.4(29.7–37.4)	27.6(17.4–33.1)	25.9(17.0–29.9)	<0.001
Procalcitonin, median (IQR) ng/mL	2.40(0.80–7.30)	1.00(0.51–2.64)	1.02(0.32–2.84)	0.18
Serum creatinine, median (IQR) mg/dL	0.85(0.49–1.19)	0.75(0.50–1.30)	0.69(0.45–1.07)	0.52
Serum lactate, median (IQR) mmol/L	1.65(1.42–2.17)	1.81(1.43–2.40)	1.50(1.23–2.13)	0.04
FiO_2_, median (IQR) %O_2_	40(30–50)	50(45–70)	50(40–70)	<0.001
SO_2_/FiO_2_, median (IQR)	261(194–322)	188(142–218)	190(149–242)	<0.001
Mean arterial pressure, median (IQR) mmHg	79(76–84)	78(73–83)	81(75–85)	0.48
Respiratory rate, median (IQR) by minute	21(17–23)	21(18–23)	20(18–22)	0.29
Heart rate, median (IQR) by minute	91(86–102)	88(77–98)	85(74–101)	0.13
Temperature, median (IQR) °C	37.9(37.2–38.3)	37.7(37.2–38.2)	37.5(37.0–37.9)	0.051
Urinary output, median (IQR) mL/24 h	1600(1300–2250)	1700(1400–2440)	1700(1300–2270)	0.16
Antibiotic empiric treatment				
Ertapenem, *n* (%)	3(6.4)	24(25.0)	24(31.2)	<0.001
Meropenem	18(38.3)	29(30.2)	43(55.8)	
Piperacillin/tazobactam	26(55.3)	43(44.8)	10(13.0)	
Adequate empirical antibiotic, *n* (%)	45(95.7)	93(96.9)	72(93.5)	0.54
Antibiotic stewardship, *n* (%)				0.63
No change	10(21.3)	25(26.0)	22(28.6)	
Desescalation	29(61.7)	50(52.1)	38(49.4)	
Escalation	7(14.9)	14(14.6)	10(13.0)	
Withdrawal/reduction	0 (0.0)	6(6.2)	6(7.8)	
Resistant microorganism	1(2.13)	1(1.0)	1(1.3)	
Bacteremic VAP, *n* (%)	11(23.4)	25(26.0)	18(23.4)	0.9
Outcome				
Mechanical ventilation days, median (IQR)	4.1(4.9–11.6)	9.3(6.0–16.1)	9.1(6.4–12.0)	0.09
ICU Length of stay, median (IQR), days	28.7(20.7–42.5)	40.8(26.9–68.4)	33.7(20.3–48.1)	0.001
Crude ICU nortality, *n* (%)	21(44.7)	36(37.5)	17(22.1)	0.02

Abbreviations: IQR = interquartile range; FiO_2_ = inspired fraction of oxygen; SO_2_: oxygen saturation, VAP = ventilator-associated pneumonia, ICU = intensive care medicine.

**Table 2:** Microorganisms isolated in respiratory samples according to the number of patients in each study period.

Microorganisms	1st Period (Patients *n* = 47) *n* (%)	2nd Period (Patients *n* = 96) *n* (%)	3rd Period (Patients *n* = 77) *n* (%)
Gram-negative bacilli			
*Pseudomonas aeruginosa*	11 (23.5)	24 (34.8)	27 (35.0)
*Serratia marcescens*	3 (6.4)	12 (12.5)	3 (3.9)
*Klebsiella* spp.	9 (19.1)	28 (29.2)	23 (29.8)
*Escherichia coli*	8 (17.0)	13 (13.5)	7 (9.0)
*Haemophilus influenzae*	3 (6.4)	10 (10.4)	10 (13.0)
*Acinetobacter baumannii*	1 (2.1)	1 (1.0)	2 (2.6)
*Enterobacter* spp.	7 (14.9)	18 (18.7)	13 (16.9)
*Proteus mirabilis*	5 (10.6)	5 (5.2)	8 (10.4)
*Stenotrophomonas maltophilia*	4 (8.5)	2 (2.0)	1 (1.3)
*Citrobacter freundii*	1 (2.1)	6 (6.2)	3 (3.9)
*Morganella morganii*	0 (0)	0 (0)	1 (1.3)
*Achromobacter xylosoxidans*	0 (0)	0 (0)	1 (1.3)
*Shewanella putrefaciens*	0 (0)	0 (0)	1 (1.3)
*Eikenella corrodens*	0 (0)	0 (0)	1 (1.3)
Gram-positive cocci			
*Staphylococcus aureus*	13 (31.9)	38 (40.6)	26 (35.0)
Methicillin-resistant *Staphylococcus aureus*	2 (4.2)	1 (1.0)	1 (1.3)
*Streptococcus anginosus*	0 (0)	3 (3.1)	2 (2.6)
*Streptococcus pneumoniae*	1 (2.1)	2 (2.0)	0 (0)
Gram-positive bacilli			
*Corynebacterium* spp.	1 (2.1)	3 (3.1)	0 (0)
Total number of isolated	69	166	130

**Table 3:** Characteristics of 220 patients according to outcome.

Variable	Survival (n = 146)	Non-Survival (n = 74)	*p*-Value
General			
Age median (IQR) year	57 (45–68)	67 (61–73)	<0.001
Male, *n* (%)	116 (79.5)	50 (67.6)	0.07
SOFA score, median (IQR)	4 (2–6)	6 (4–8)	<0.001
Medical disease, *n* (%)	108 (74.0)	55 (74.3)	1.0
COVID-19, *n* (%)	60 (41.1)	36 (48.6)	0.35
Filmarray, *n* (%)	120 (82.2)	53 (71.6)	0.10
Major comorbidities			
Diabetes mellitus, *n* (%)	28 (19.2)	21 (28.4)	0.16
Cirrhosis, *n* (%)	11(7.5)	5 (6.7)	1.0
Hypertension. *n* (%)	65 (44.5)	34 (45.9)	0.9
Chronic heart disease, *n* (%)	1 (0.7)	5 (6.7)	0.01
Immunodepression, *n* (%)	10 (6.8)	9 (12.2)	0.28
Chronic obstrcutive pulmonary disease, *n* (%)	17 (11.6)	10 (13.5)	0.7
Laboratory at ICU admission			
Hemoglobin, median (IQR) g/L	8.9 (7.9–10.3)	8.8 (7.8–9.9)	0.54
White blood cells count, median (IQR) 10 × 9	14.7 (10.3–19.3)	14.4 (9.3–21.2)	0.78
Lymphocytes, median (IQR) 10 × 9	0.9 (0.5–1.2)	0.7 (0.4–1.1)	0.07
C-reactive protein, median (IQR) mg/dL	28.3 (18.9–34.0)	29.0 (18.0–34.1)	0.57
Procalcitonin, median (IQR) ng/mL	0.86 (0.33–2.77)	1.35 (0.67–4.47)	0.02
Serum creatinine, median (IQR) mg/dL	0.71 (0.45–1.04)	0.87 (0.52–1.52)	0.06
Serum lactate, median (IQR) mmol/L	1.58 (1.32–2.15)	1.76 (1.43–2.46)	0.15
Clinical variables			
FiO_2_, median (IQR) %O_2_	50 (40–60)	50 (40–70)	0.05
SO_2_/FiO_2_, median (IQR)	200 (162–249)	188 (144–242)	0.04
Mean arterial pressure, median (IQR) mmHg	81 (76–85)	76 (72–82)	0.001
Respiratory rate, median(IQR) by minute	21 (18–23)	20 (18–23)	0.62
Heart rate, median (IQR) by minute	86 (76–99)	87 (79–101)	0.20
Temperature, median (IQR) °C	37.7 (37.2–38.2)	37.5 (37.0–38.2)	0.10
Urinary output, median (IQR) mL/24 h	1900 (1400–2400)	1500 (100–2200)	0.007
Empiric treatment			
Ertapenem, *n* (%)	34 (23.3)	17 (23.0)	0.54
Meropenem, *n* (%)	63 (43.2)	27 (36.5)	
Piperacillin/Tazobactam, *n* (%)	49 (33.6)	30 (40.5)	
Adequate empiric antibiotic treatment, *n* (%)	140 (95.9)	70 (90.6)	0.73
Outcome			
Bacteremic VAP, *n* (%)	35 (24.0)	19 (25.7)	0.91
Mechanical ventilation days, median (IQR)	9 (6–13)	8 (5.7–13)	0.93
ICU length of stay, median (IQR), days	43 (28–58)	24 (16–37)	<0.001

Abbreviations: IQR = interquartile range; FiO_2_ = inspired fraction of oxygen; SO_2_: oxygen saturation, VAP = ventilator-associated pneumonia, ICU = intensive care medicine.

## Data Availability

The data supporting the conclusions of this study are available from the Joan XXIII de Tarragona Hospital (Spain), but restrictions are placed on the free availability of these data by the health authorities of Catalonia, so they are not publicly available. However, the data can be obtained from the corresponding author (A.R.) upon reasonable request and with the permission of the Technical Secretary and the person responsible for data management at Joan XXIII de Tarragona Hospital (Spain).

## Supplementary Materials

The following supporting information can be downloaded at https://www.mdpi.com/article/10.3390/biomedicines13020360/s1. Figure S1: Flow Chart of included patients according to period study; Figure S2: Incidence density of ventilator-associated pneumonia /1000 ventilator days according to controlled periods; Figure S3: Area under the ROC curve for the prediction of the multivariate mortality model; Table S1: Micro-organisms isolated and empirical treatment in the 10 patients with inappropriate empirical antibiotic treatment (IEAT).; Table S2: Cross-validation results (K = 10).
