# Supplementary material for: Ten-Year Evaluation of Ventilator-Associated Pneumonia (VAP) According to Initial Empiric Treatment: A Retrospective Analysis Using Real-World Data
Source: Biomedicines. 2025 Feb 5;13(2):360. doi: 10.3390/biomedicines13020360 (PMC11852478; doi:10.3390/biomedicines13020360)
Supplement: Supplementary file 1 [file biomedicines-13-00360-s001.zip › biomedicines-3421610-supplementary.pdf]

Ten-year evaluation of ventilator-associated pneumonia (VAP) according to initial empiric treatment. A retrospective analysis using real-world data.

Supplementary material

Figure S1: Flow Chart of included patients according to period study

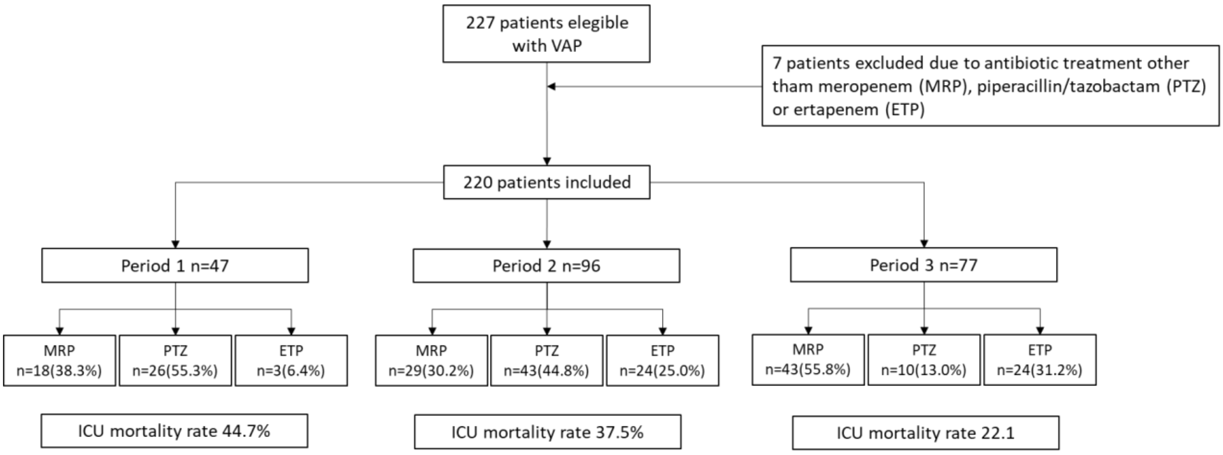

Figure S2: Incidence density of ventilator-associated pneumonia /1000 ventilator days according to controlled periods

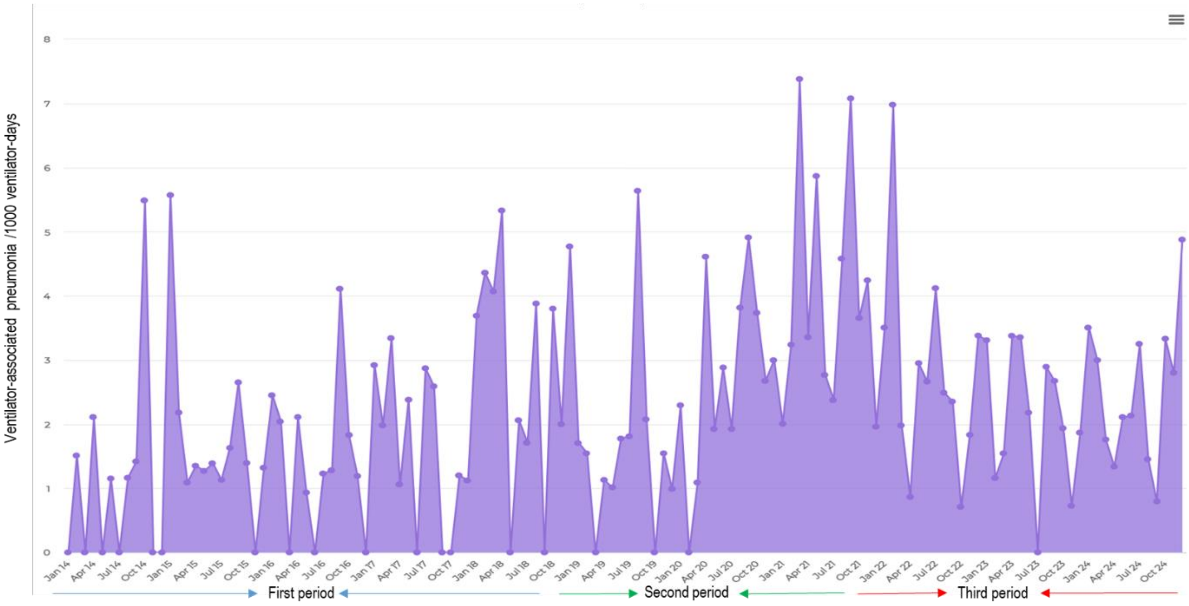

Table S1: Micro-organisms isolated and empirical treatment in the 10 patients with inappropriate empirical antibiotic treatment (IEAT)

Patient number	Microorganims isolated	IEAT
2	*Stenotrophomonas maltophilia*	Ertapenem
3	*Pseudomonas aeruginosa* VIM	Meropenem
4	*Stenotrophomonas maltophilia* + *Pseudomonas aeruginosa* MDR	Meropenem
17	*Stenotrophomonas maltophilia* + *Pseudomonas aeruginosa* MDR	Piperacillin/Tazobactam
19	*Pseudomonas aeruginosa* MDR + *Klebsiella pneumoniae*	Piperacillin/Tazobactam
20	Methicillin-resistant *Staphylococcus aureus*	Ertapenem
21	*Pseudomonas aeruginosa* VIM	Piperacillin/Tazobactam
169	*Stenotrophomonas maltophilia* + *Klebsiella pneumoniae OXA-48*	Piperacillin/Tazobactam
170	*Stenotrophomonas maltophilia*	Piperacillin/Tazobactam
171	*Stenotrophomonas maltophiliaE*	Meropenem

Figure S3: Area under the ROC curve for the prediction of the multivariate mortality model.

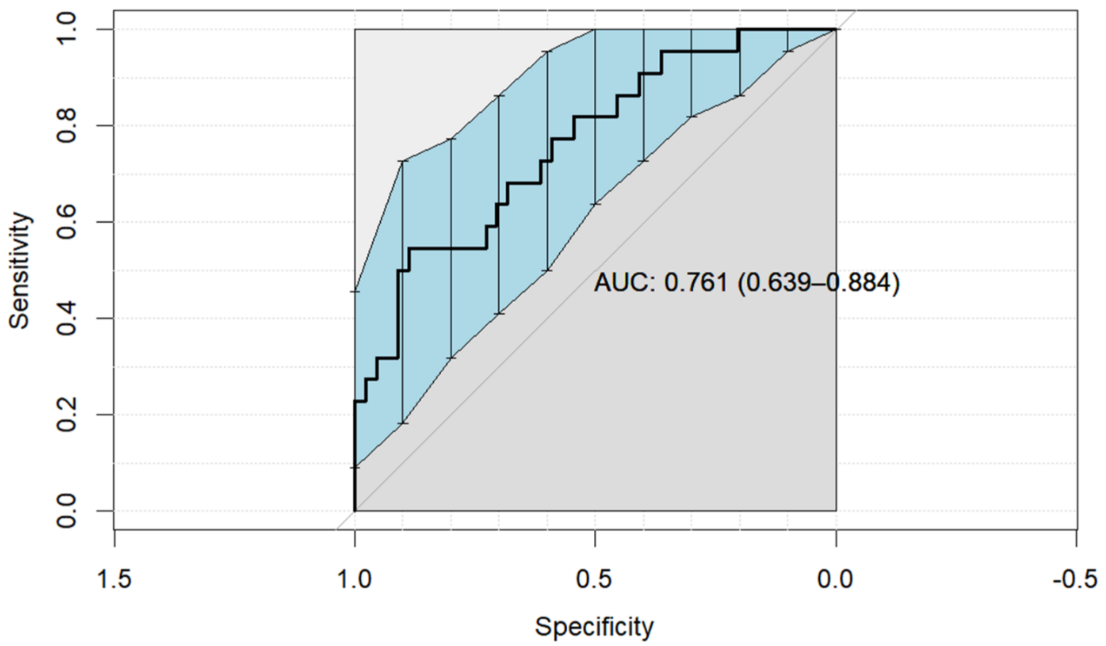

Table S2: Cross-validation results (K=10)

```
Confusion Matrix and Statistics

          Reference
Prediction  0  1
         0 37 10
         1  7 12

               Accuracy : 0.7424
                 95% CI : (0.6199, 0.8422)
    No Information Rate : 0.6667
    P-Value [Acc > NIR] : 0.1187

                  Kappa : 0.4

 Mcnemar's Test P-Value : 0.6276

            Sensitivity : 0.8409
            Specificity : 0.5455
         Pos Pred Value : 0.7872
         Neg Pred Value : 0.6316
             Prevalence : 0.6667
         Detection Rate : 0.5606
   Detection Prevalence : 0.7121
      Balanced Accuracy : 0.6932

       'Positive' Class : 0
```
